# Rehabilitation of the Atrophic Edentulous Maxilla: A Retrospective Cohort Study Comparing Survival of Delayed‐Loaded Implants in Grafted Bone Versus Immediately Loaded Implants in Native Bone

**DOI:** 10.1002/cre2.70167

**Published:** 2025-11-18

**Authors:** Evelina Haroyan‐Darbinyan, Qiman Gao, Pablo de Lillo, Jesús Torres, Samer Abi‐Nader, Daniel Nach, Faleh Tamimi

**Affiliations:** ^1^ Faculty of Dental Medicine and Oral Health Sciences McGill University, Strathcona Anatomy and Dentistry Building Montreal Quebec Canada; ^2^ Faculty of Statistics Complutense University of Madrid (U.C.M) Madrid Comunidad de Madrid Spain; ^3^ Faculty of Dentistry Complutense University of Madrid (U.C.M) Madrid Comunidad de Madrid Spain; ^4^ East Coast Oral Surgery Moncton New Brunswick Canada; ^5^ College of Dental Medicine. College of Dental Medicine, QU Health Qatar University Doha Ad‐Dawha Qatar

**Keywords:** bone regeneration, implant survival, sinus floor augmentation, tilted implants

## Abstract

**Objectives:**

This retrospective cohort study aimed to assess the survival rate of implants placed in grafted edentulous maxillary arches following a delayed loading protocol versus a graftless approach with an immediate loading protocol.

**Materials and Methods:**

Eighty seven patients with atrophic edentulous maxillae were included in two groups: Group‐1 (GG group, *n* = 155 implants): 26 patients that underwent maxillary bone grafting before treatment with axially placed delayed loading implants and provided with a fixed full‐arch prostheses; Group‐2 (GL group; *n* = 244 implants): 61 patients who received axial and tilted implants without bone augmentation followed by an immediately loaded fixed full‐arch prostheses. Patients were followed up for up to 10 years. Kaplan–Meier and Mantel–Cox analyses were performed to determine implant survival rates, and a Cox hazards model was run to assess the influence of patient, implant, and prosthesis‐based covariates.

**Results:**

There were no significant differences in implant failure rates between the two treatment groups (p = 0.298). Five implant failures were observed in Group‐1 (GG group) and four failures were observed in Group‐2 (GL group) (*N* = 9). Survival rate was 96.8% and 98.4% in the GG and GL groups, respectively. No significant association between patient and implant‐based covariates and implant failure was observed in both groups; however, a significant association was observed regarding the nature of the opposing arch (p = 0.019).

**Conclusion:**

Immediately loaded implants placed in maxillary native bone show statistically similar survival rates compared to implants placed in grafted bone following a delayed loading. The nature of the opposing arch may negatively influence the survival rate of dental implants.

**Clinical Significance:**

For atrophic edentulous maxillae, both grafted and graftless approach may represent a viable treatment modality in the long term.

## 1 Introduction

Rehabilitation of the atrophic edentulous maxilla presents many surgical and restorative challenges due to anatomical limitations and poor bone quality despite the wide variety of surgical procedures available (Aghaloo et al. 2017; Morand and Irinakis 2007). Traditionally, in cases where the patient is looking for a fixed prosthesis, creation of sufficient bone volume to embed an implant with an adequate length and diameter has been mandatory (Aghaloo et al. 2017). In this sense, the treatment of the atrophic maxilla was delivered using maxillary sinus floor augmentation (MSFA) combined with onlay grafting of the alveolar process. This step is followed by implant placement using a delayed loading protocol (Peñarrocha‐Oltra, Candel‐Martí, et al. 2013) and the delivery of a fixed prosthesis once the osseointegration is achieved. However, despite its high success rates and reliable long‐term results (Jemt and Johansson 2006; Raghoebar et al. 2019), grafting of the atrophic maxilla requires multiple surgical interventions (Peñarrocha‐Oltra, Candel‐Martí, et al. 2013), and increases the surgical morbidity (Del Fabbro et al. 2004) and the overall cost of the treatment (Babbush et al. 2014). In addition, the transition period is very challenging for the patients, as they often need to wear a removable transitional rehabilitation or remain completely edentulous for an extended period of time before receiving the final fixed prosthesis.

Various treatment options have been described in the literature to manage the severely atrophic edentulous maxilla without the use of bone grafting, including short implants (Monje Fu, et al. 2013; Monje, Chan, et al. 2013), tilted implants (Aparicio et al. 2001; Hamilton et al. 2021), zygomatic implants (Aparicio et al. 2014), pterygoid implants (Araujo et al. 2019), and the All‐on‐Four® protocol (Maló et al. 2003, 2005), among others. These alternative treatment protocols offer patients a significant reduction in total treatment time and cost, and most importantly, the benefit of immediate delivery of a transitional fixed prosthesis following implant placement (Al‐Sawai and Labib 2016; Crespi et al. 2012). The All‐on‐Four® technique was first introduced by Maló (Maló et al. 2003) in 2003 to reduce the number of implants, treatment time, and costs as well as to offer patients and clinicians a smooth transitional period until the delivery of the final prosthesis (Maló et al. 2005; Al‐Sawai and Labib 2016; Crespi et al. 2012). This procedure involves the use of four implants: the two most anterior implants are inserted axially while the two posterior implants are distally tilted to minimize the length of the prosthetic cantilever (Menendez‐Collar et al. 2018; Maló et al. 2019, 2015), hence improving load distribution (Lin and Eckert 2018; Casar‐Espinosa et al. 2017).

On the one hand, the All‐on‐Four® treatment concept enables the clinician to provide patients with an immediately loaded full‐arch fixed prosthesis supported by four implants on the day of surgery (Maló et al. 2003), increasing the patients’ quality of life while providing a substantial financial benefit (Gonçalves et al. 2022). On the other hand, retrospective studies on the All‐on‐Four® concept yielded an accumulative survival rate of 97.4% by implant level for the maxillae with up to 17 years follow‐up (Maló et al. 2019; Uesugi et al. 2023; Agliardi et al. 2023). However, despite the current growing number of studies on implant survival in the atrophic maxilla, there is no consensus or robust data comparing implant success between the graft treatment approach and the graftless approach in restoring the atrophic edentulous maxillae (Tealdo et al. 2008; Balshi et al. 2005).

Therefore, the aim of this retrospective cohort study was to compare the survival rate of conventional dental implants placed in regenerated maxillary bone sites following a delayed loading protocol versus implants placed following the graftless approach with an immediate loading protocol in moderate‐to‐severe atrophic edentulous maxillae. Thus, the null hypothesis tested was that restoring the atrophic edentulous maxillae with a grafted approach following delayed‐loading implants and graftless All‐on‐Four®All‐on‐Four® technique shows no significant differences from each other concerning the survival rate of implants.

## 2 Material and Methods

### 2.1 Study Design

This retrospective cohort study was carried out in accordance with the Declaration of Helsinki guidelines and was approved by the Research Ethics Committee for Clinical Trial of McGill University (12‐321 GEN). The study was designed and reported in accordance with the Strengthening the Reporting of Observational Studies in Epidemiology (STROBE) guidelines (Cawood and Howell 1988).

### 2.2 Study Population and Data Source

**Figure 1:**
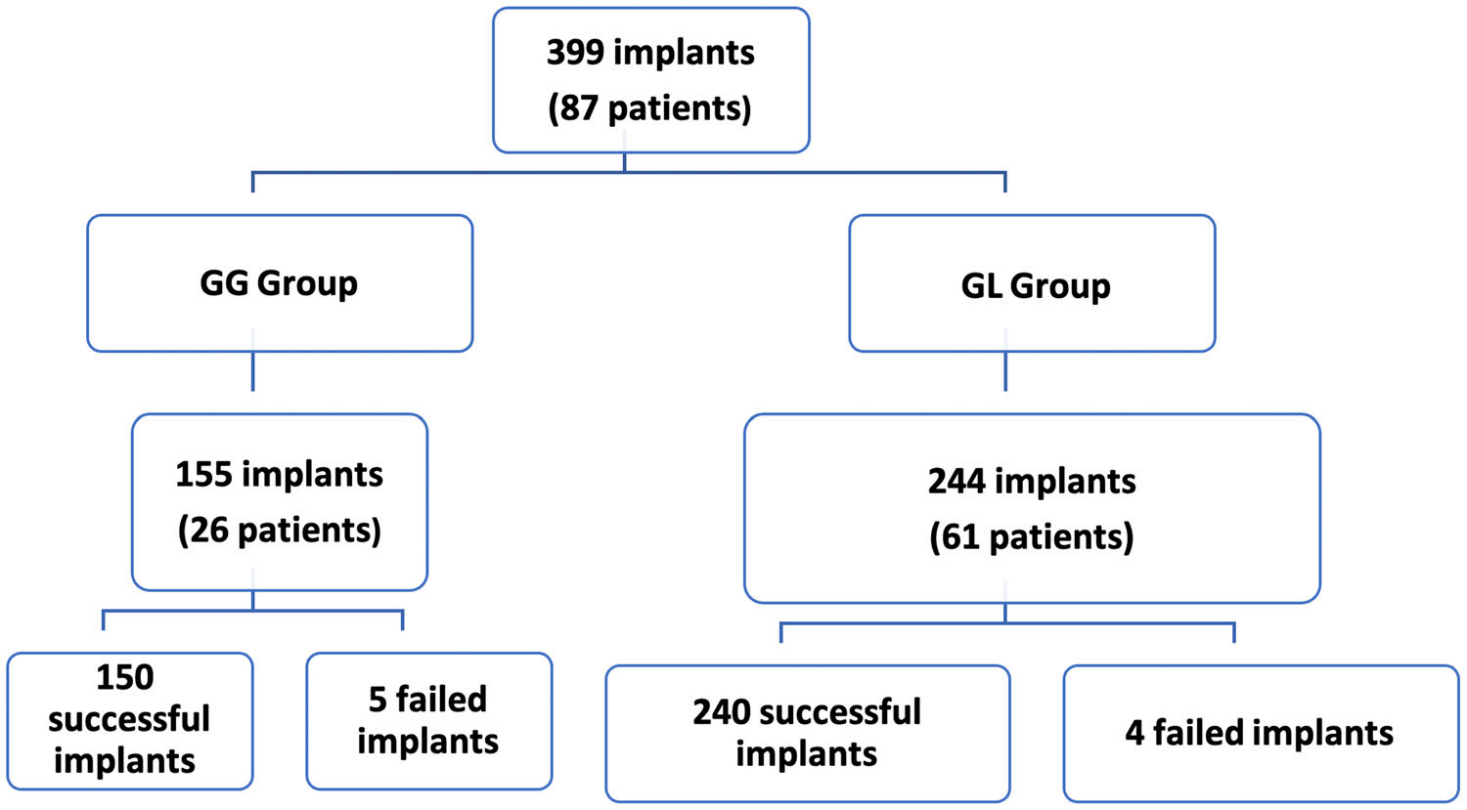
Multiplanar cone‐beam computed tomography (CBCT) slices of a patient rehabilitated according to the All‐on‐Four® treatment concept in the edentulous maxillae. A: Axial cut showing antral floor. B: Frontal cut illustrating the All‐on‐Four® approach. C: 3D digital reconstruction of a severely resorbed edentulous maxillae. D: Sagittal cut illustrating the implant placement.

The study cohort consisted of the medical records of patients who received a prosthodontic rehabilitation of a completely edentulous maxillae from January 2007 to September 2016 at a private clinic (East Coast Oral Surgery, Moncton, New Brunswick, Canada). A total of 87 patients (40 males, 47 females) (Figure 1) were included in two treatment modalities: the grafted group (GG) and graftless group (GL). The GG group included 26 patients (9 males, 17 females) who received a full‐arch fixed implant‐supported prosthesis following bone grafting of the maxilla and a delayed loading protocol (Peñarrocha‐Oltra, Covani, et al. 2013). The GL group was comprised of 61 patients (31 males and 30 females) who were treated by maxillary fixed implant‐supported prostheses following an immediate loading protocol (conventional axial and tilted implants).

### 2.3 Preoperative Assessment and Preparation

Patients first received an extraoral/intraoral examination, and a standardized questionnaire was used to record social demographic information (age, sex), smoking habits, systemic diseases, and use of medication. Before the treatment, participants were informed of the risks and benefits as well as the necessary information on the duration and costs for each proposed treatment modality. All patients were treated by a single surgical and restorative team after signing an informed consent.

The enrollment criteria comprised: (1) patients aged at least 20 years at time of surgery; (2) moderate to severe atrophic edentulous maxillae (class III to VI, following Cawood and Howell classification of edentulous jaws) (Cawood and Howell 1988) with pneumatization of the maxillary sinus requiring sinus floor elevation procedures for implant placement purposes; (3) Medically fit patients (free of any systemic or local diseases that might contraindicate the placement of dental implants). The exclusion criteria were signs and symptoms of maxillary sinus disease and systemic diseases that could influence implant osseointegration.

All patients received a Cone‐Beam Computed Tomography scan (CBCT) (iCAT FLX, Henry Schein®, Melville, NY, USA) for preoperative assessment. Patients were allocated for treatment with the grafted or graftless approach through a shared decision with each patient. This involved assessment of the residual ridge condition, the bone quality, and the patients’ preferences after discussing with them the perceived benefits and risks of each treatment option.

Patients were recommended for the maxillary bone augmentation and delayed protocol (GG group) in cases of severe maxillary atrophy (Classes IV to VI, according to Cawood and Howell classification (Cawood and Howell 1988)) with presence of sinus pneumatization associated with an unfavorable intermaxillary relationship in the vertical, anteroposterior and lateral axes of the sagittal and coronal planes (Cawood and Howell 1988; Chiapasco and Zaniboni 2009). Severe maxillary bone resorption was defined as knife‐edge ridge form, adequate in height and inadequate in width (class IV); flat ridge form, inadequate in height and width (Class V); and depressed ridge form, with some basilar loss evident (Class IV) (Cawood and Howell 1988).

Conversely, patients were recommended for the immediate‐loading protocol (GL group), when sinus pneumatization was relevant but there was sufficient bone volume to embed two tilted implants in the posterior ridge and two axial implants in the anterior ridge that were at least 4‐mm in diameter and 10‐mm in length (Cawood and Howell 1988).

### 2.4 Surgical Procedure

All surgical treatments (bone grafting and implant placement) were provided by the same surgeon (D.N.), thus eliminating any bias related to personal experience, technique, or skill.

On the one hand, in the GG group, the atrophied maxillary bone was managed with autologous bone grafts, and patients received conventional axial implants 6–9 months following the grafting procedure. The grafting procedure was performed under general anesthesia. Autogenous bone (cortico‐cancellous blocks) was collected from the iliac crest to graft the maxilla (Naenni et al. 2019). Tricortical wedges from the anterior or the posterior iliac crest were shaped and used as an onlay graft in the alveolar area. The grafted bone was fixated using osseosynthesis screws (2.0 mm diameter, 10 mm length) (DePuy Synthes, Johnson and Johnson, NJ, USA). Before fixing the onlay grafts, a sinus floor lift procedure was performed in the posterior maxilla as previously described by Tatum (Tatum 1986). Autogenous cancellous bone collected from the iliac crest was combined with allogeneic cortico‐cancellous bone (from the hospital bone bank) and used to graft the maxillary sinus cavities (Naenni et al. 2019). Bioabsorbable collagen membranes (Bio‐Guide, Geistlich Biomaterials, Wolhusen, Switzerland) were used to cover the grafted bone before closure of the flap and for repair of the sinus membrane in cases where it was ruptured. Patients were instructed not to wear their transitional complete denture for 7–10 days after the surgery, which was adjusted and relined at the check‐up appointment. After 6 months of healing, a second CBCT was obtained to assess bone availability to insert the necessary implants. Surgical planning for the implant placement of all cases was completed using the NobelGuide and DTX Studio (Nobel Biocare, Kloten, Switzerland). All implants in the GG group were Biomet 3i with acid‐etched surface (Osseotite, Biomet, West Palm Beach, FL, USA) or Nobel Biocare with moderately rough anodized (TiO₂) surface (TiUnite, Nobel Biocare, Kloten, Switzerland). All grafted group patients received delayed‐loading implants. Four to seven implants were placed and buried following the original Bränemark two‐stage protocol (Buser et al. 2017). Six months later, the implants were uncovered, and the prosthetic phase for delivery of the final fixed prosthesis was initiated.

**Figure 2:**
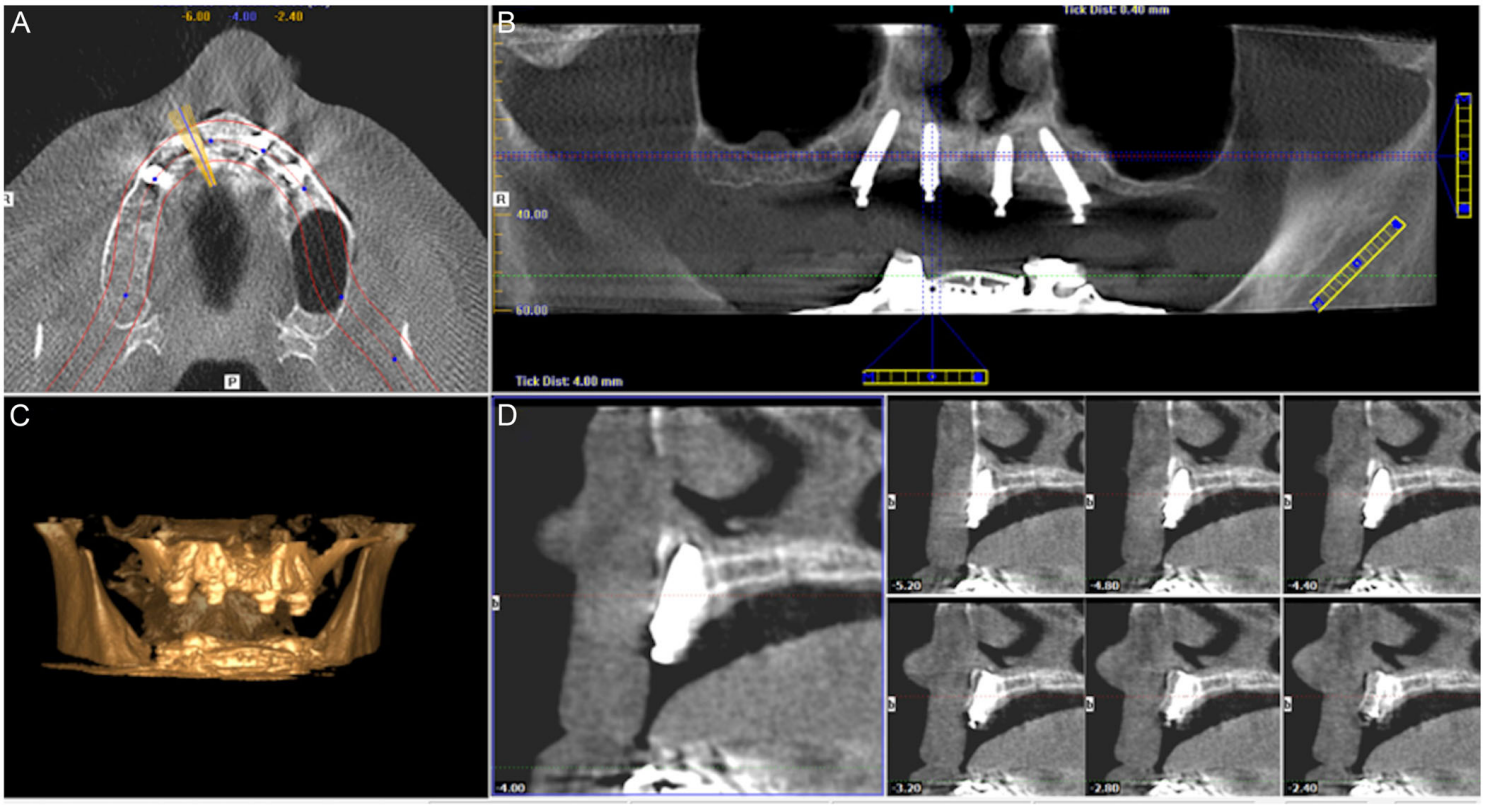
Flow diagram of participants in each group (patients/implants).

On another hand, all patients in the GL group received immediately loaded implants supplied by Nobel Biocare with Tiunite surface (Kloten, Switzerland), following the standard All‐on‐Four® procedure (Figure 2), when sufficient bone volume was available according to the Cawood and Howell classification (Cawood and Howell 1988) by means of a CBCT. Axial implants were placed in the area corresponding to the middle and lateral incisors. Distally tilted implants in the posterior region were positioned just anterior to the maxillary sinus cavity. The drilling procedure was completed according to Malo's protocol (Maló et al. 2005, 2015).

### 2.5 Postoperative Evaluation

Postoperatively, clinical parameters such as implant length, implant diameter, insertion torque, and bone augmentation were recorded. Patients of both groups received a prescription of antibiotics for a period of 7 days: Amoxicillin 500 mg, orally, three times per day (Amoxicillin Sanis, Sanis Health Inc, Brampton, Ontario, Canada) or in case of allergy, Clindamycin 300 mg, orally, four times per day (Clindamycin Sanis, Sanis Health Inc). Analgesic agents were prescribed as needed using Tylenol (500 mg, t.d.s.; Johnson & Johnson, NJ, USA) or Advil (400 mg, t.d.s.; Wyeth Consumer Healthcare, Madison, NJ, USA). Oral hygiene instructions were provided. Patients were instructed to rinse three to four times per day with 0.12% chlorhexidine solution (Peridex, Periogard, Allentown, PA, USA) for the first 7–10 days following the procedure. They were also instructed to keep a soft diet for the first 72 h, followed by a semi‐solid diet for the next 3 months. Patients were recalled for follow‐up examinations and removal of sutures 10 days after surgery. All patients were scheduled for postoperative surgical assessment at 10 days, 4 weeks, and 6 months following implant placement, and/or whenever they encountered a complication with their implants. Any unusual condition they experienced was recorded.

### 2.6 Prosthodontic Procedure and Follow‐Up

All restorative treatments were delivered by the same restorative dentist (SAN); therefore, any bias related to personal experience, technique, or skill was eliminated.

For the graftless group (GL), straight or angled Multiunit® abutments (Nobel BioCare®, Kloten, Switzerland) were placed to achieve relative parallelism of the implants to achieve a rigid prosthetic framework and a passive fit.

Immediately after surgery, a pick‐up impression using the patient's existing complete maxillary prostheses was performed by means of polysiloxane material (Virtual, Ivoclar‐Vivadent, Schaan, Liechtensetin), and a model was poured with quick stone following the recommended guidelines (ISO Type 3, Whipmix, Louisville, KY, USA). A transitional all‐acrylic full‐arch screw‐retained prosthesis was fabricated and delivered. The fitting surfaces of the prosthesis were designed to be accessible, cleanable, and in intimate contact with the soft tissues of the edentulous ridge. The prosthesis was fitted and tightened by the prosthetic screws at 15 N/cm (Nobel Manual Torque Wrench, Nobel BioCare). The occlusion was adjusted to provide bilateral contacts in maximum intercuspation while occlusal contacts on the posterior cantilevers were avoided during the osteointegration period. All prostheses were fabricated and delivered within 3–4 h after completion of the surgery. The screw access was covered with a light cured temporary composite material (Fermit‐N, Ivoclar‐Vivadent, Schaan, Liechtensetin).

Patients in the GG group were examined 10 days postoperatively and instructed to clean the provisional prostheses and alveolar ridge once a day with an unwaxed dental floss (Johnson & Johnson, NJ, USA) and a floss threader (GUM Eez‐Thru Floss, Sunstar Americas, Chicago, IL, USA). They were also instructed to brush the prostheses and alveolar ridge three times a day with a soft toothbrush (GUM Delicate Postsurgical Toothbrush, Oral B–Gillette, Redwood, CA, USA) and a low‐abrasive toothpaste (DentuCream, Block Drug Co., Jersey City, NJ, USA).

Four to six months after initial loading, the transitional prostheses were removed and evaluated clinically by assessing vertical, lateral, and rotational signs of mobility (Soave and Sun 2017), and a periapical radiograph was taken when in doubt. A final impression of the maxillary implants was completed using an elastomeric material: putty polyvinyl siloxane and heavy body polyvinyl siloxane (Virtual, Ivoclar‐Vivadent, Schaan, Liechtensetin). The impression copings were splinted intraorally using a self‐curing acrylic material (Pattern Resin LS, GC America) before the final impression procedure to minimize any risk of movement during the impression technique. A final full‐arch screw retained prosthesis with titanium prosthetic frame coated with acrylic resin and acrylic artificial teeth was fabricated using the Nobel BioCare Procera abutment system and delivered to the patients following the All‐on‐Four treatment protocol (Ho et al. 2013).

For the GG group with conventional implants, a final full‐arch screw‐retained prostheses were fabricated with a titanium framework, acrylic veneering, and acrylic teeth following a similar prosthetic protocol as described above.

### 2.7 Study Outcomes and Statistical Analysis

In the routine follow‐up protocol of the dental clinic, patients with implant‐supported prostheses are seen for clinical examination every year; a panoramic X‐ray is performed, and clinical findings are documented in the patient's file. The primary outcome of this study was to assess the implant survival rate in the two cohorts; hence, the unit of analysis was the implant. Implant failure was determined according to the International Congress of Oral Implantologists (ICOI) consensus of 2008 (Misch et al. 2008). Implants exhibiting (a) pain on function; (b) mobility; (c) radiographic bone loss equivalent to 1/2 of the implant length; (d) uncontrolled exudate; and (e) implant no longer in the mouth were categorized as failures. Patient‐based demographic parameters (age, sex, smoking), implant‐based demographics (implant dimensions, insertion torque, angulation, and loading protocols), and prosthetic information were collected.

The data were analyzed using the statistical software Stata 16 (Stata Corp, College Station, Texas, USA). Descriptive statistics were reported as means with standard deviations (SD) and the frequencies were expressed as percentages. As the Shapiro‐Wilk test confirmed the normality of distributions, parametric probes were selected (Vetter 2017). Levene's test was performed to assess the equality of variances (Soave and Sun 2017). Student's *t*‐test was used to assess the difference between the two groups (GG and GL) in terms of patient or implant‐based demographics.

Implant failure distribution was evaluated using a time‐to‐event analysis. Kaplan–Meier survival curves were plotted, and Log‐Rank Mantel‐Cox tests were used to compare cumulative survival rates (Rich et al. 2010). Since all patients had multiple implants, the possibility of a cluster behavior of implant failure among the patients was considered. The Cox proportional‐hazards model with clustered data analysis was run to estimate the effect of patient and implant‐based covariates on survival time (Bender 2009). The following confounders were adjusted in the Cox regression model with clustered data: age and sex of the patients, smoking habit, diameter, length, and torque of the implants, angulation of the implants, presence of grafting, type of implant (axial or tilted), implant site, number of implants placed, and the nature of the opposing arch to the implants. The level of significance was set in advance at *α* = 0.05. Survival rate of the implants according to implant angulation and loading protocols was also assessed as a secondary analysis.

### 2.8 Power and Sample Size Estimation

A *post‐hoc* retrospective power analysis was performed to examine if the results had enough power (Zhang et al. 2019). According to Lachin's method (Lachin 1981), based on the total number of implants included at the final time point (*n* = 404) and the failure events (*n* = 9), the study had a power of 87.37% at a 5% one‐side significance level. The power of this study was enough to detect the difference in implant survival rate between the grafted and graftless groups over the up to three‐year follow‐up period.

## 3 Results

### 3.1 Case Flow

From January 2007 to September 2016, a total of 87 patients with edentulous maxillae, 47 (54.0%) males and 40 (46.0%) females, were rehabilitated with a fixed full‐arch screw‐retained maxillary prostheses (Table 1, Figure 1). A total of 399 implants were placed and included in the analysis: patient‐based demographics in each group are shown in Table 1.

**Table 1:** Patient‐based demographics of grafted (GG) and graftless (GL) cases.

	GL group	GG group	*p*‐value
*N* patients	61	26	
*N* total implants	244 (61.2%)	155 (38.8%)	
*N* implants/patient [mean (SD)]	4.0 (0.4)	6.1 (0.7)	< 0.001^a^
*N* posterior implants/patient [mean (SD)]	1.9 (0.5)	3.9 (0.5)	< 0.001^a^
Patient age, surgery moment [mean (SD)]	61.1 (8.2)	58.2 (9.1)	< 0.001^b^
Follow‐up period [mean (SD)] months	92.5 (29.8)	99.3 (32.8)	0.032^b^
Time to function [mean (SD)] months	0.64 (1.81)	19.14 (6.91)	< 0.001^a^
Sex			0.165^c^
Females [*n* (%)]	31 (50.8)	9 (34.6)	
Males [*n* (%)]	30 (49.2)	17 (65.4)	
Smokers [*n* (%)]	11 (18.0)	0 (0)	0.029^d^

^a^ Student *t* test, equal variances not assumed.

^b^ Student *t* test, equal variances assumed.

^c^ Pearson *χ* ^2^.

^d^ Fisher's exact test.

A total of 26 patients in the GG group received a maxillary fixed prosthesis supported by delayed‐loaded implants placed on augmented bone while in the GL group, 61 patients received a maxillary fixed implant‐supported prosthesis supported by immediately loaded implants in pristine bone (Table 1).

### 3.2 Survival Rates and Kaplan−Meier Estimates

Implant‐based and prosthetic characteristics are presented in Table 2. The tested groups yielded statistically significant differences in implant length, implant torque, and number of implants (*p* < 0.001; Table 2). As for the time‐to‐function, GL group (0.62 ± 1.78 months after implant placement) presented significantly shorter period (*p* < 0.001) until functioning than the GG group (19.13 ± 6.91 months after the initial grafting procedure; Table 2).

**Table 2:** Implant‐based demographics of grafted (GG group) and graftless (GL group) cases.

	GL group (*n* implants = 244)	GG group (*n* implants = 155)	*p*‐value
Survival rate	98.4%	96.8%	0.298^a^
Implant diameter (mm), mean (SD)	4.0 (0.0)	4.08 (0.18)	< 0.001^b^
Implant length (mm), mean (SD)	13.06 (1.92)	11.43 (1.24)	< 0.001^b^
Implant torque (N·cm), mean (SD)	37.81 (12.42)	22.98 (8.91)	< 0.001^b^
Time to function (months)^c^, mean(SD)	0.62 (1.78)	19.13 (6.91)	< 0.001^b^
Number of implants, mean (SD)	4.0 (0.0)	6.1 (0.7)	< 0.001^b^
Opposing arch			0.019^d^
RCD/Overdenture	11 (4.51%)	12 (7.74%)	
Natural Dentition/IS‐FPD	201 (82.38%)	109 (70.32%)	
IS‐FFA	32 (13.11%)	34 (21.94%)	

Abbreviations: IS‐FFA, implant‐supported fixed full‐arch prostheses; IS‐FPD, implant‐supported fixed‐partial denture; RCD, removable complete denture.

^a^ Log Tank Mantel‐Cox test.

^b^ Student *t*‐test, equal variances assumed.

^c^ Mean time to function since the grafting procedure.

^d^ Holm‐Bonferroni *post‐hoc* correction after Pearson's *χ* ^2^ test.

**Figure 3:**
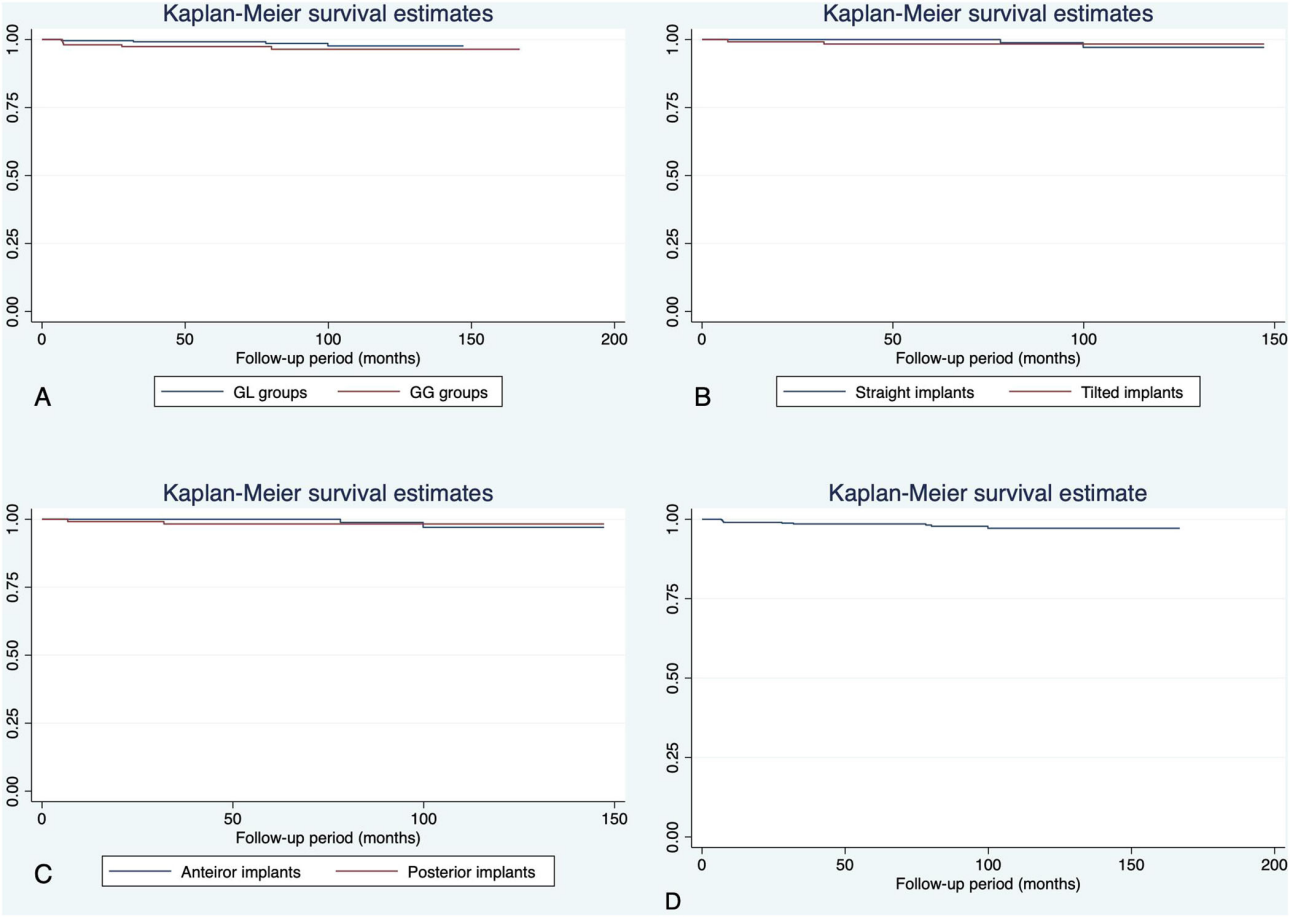
(A) Kaplan−Meier graph analysis of implant survival rate in the GL and GG groups for up to 10 years. (B) Kaplan−Meier graph analysis of straight and tilted implants within the graftless group (GL). (C) Kaplan−Meier graph analysis of anterior and posterior implants within the GL group. (D) Kaplan−Meier graph analysis of all implants.

The overall implant survival in both groups was 97.7% (390/399) (Figure 3D). There were five implant failures (5/155) in the GG group (survival rate of 96.8%; Table 2) and four implant failures (4/244) in the GL group (survival rate of 98.4%; Table 2). Log Tank Mantel–Cox test showed no statistically significant differences between the GG and the GL groups regarding implant survival rates (*p‐*value: 0.298; CI 95%: 0.59–6.92) (Figure 3A).

Within the GL group, the survival rate of the straight implants (98.4% (120/122)) was not significantly different from the survival rate of the tilted implants (98.4% (120/122)) (Mantel–Cox test *p*‐value = 0.966) (Figure 3B). Within the GL group, the survival rate of the anterior implants (98.4% (126/128)) was not significantly different from the survival rate for posterior implants (98.3% (114/116)) (Mantel–Cox test *p*‐value = 0.202). (Figure 3C). Patient age, sex, smoking habits, as well as implant dimensions, insertion torque, implant location, and angulation were not significantly associated with implant failure (Table 4).

### 3.3 Description of Failed Implants

All failed implants were in non‐smokers (Table 4), and they took place in different patients. In the GG group, one implant failed in a patient with depression, one in a patient with a previous oncogenic process and depression, one in a patient with hypercholesterolemia and hypertension, and two implants failed in patients with no systemic diseases. In this group, all failures occurred at least 7 months after implant placement. Two of the failed implants in the GG group had a length of 10.0 mm, and three implants had a length of 11.5 mm. In the GL group, one implant failure occurred after 7 months. This patient had a history of diabetes, hypertension, and hypothyroidism. The other three implant failures were observed in patients with no systemic diseases, and they occurred 2.6, 6.5, and 8 years after the implant placement, respectively. The four failed implants in the GL group had a diameter of 4 mm, and lengths of 10 mm, 11.5 mm, and two of 15‐mm.

## 4 Discussion

Adequate restoration of a severely atrophic maxillae represents a significant challenge, as conventional implant procedures are dependent on the location and volume of remaining cortical bone. For many decades, implant rehabilitation of atrophied edentulous ridges was delivered following bone grafting techniques combined with a delayed placement and loading of standard axial implants, to overcome these anatomical and physiological limitations. This traditional approach offered clinicians and patients a viable treatment solution with predictable long‐term outcomes (Shi et al. 2020; Qian et al. 2020). In spite of their predictability, the treatment mentioned above represents several disadvantages, especially high morbidity at both donor and recipient sites (Nkenke and Neukam 2014), as well as a lengthy recovery (Starch‐Jensen et al. 2021), a longer delay until delivery of the final prosthesis, and increased treatment costs, which may negatively affect the patient's quality of life (Del Fabbro et al. 2004; Starch‐Jensen et al. 2021).

Considering these limitations, another graftless therapeutic option has gained popularity in the past decade, which involves the use of tilted implants to engage dense native bone in the maxillary (Aparicio et al. 2001; Agliardi et al. 2010; Abouelhuda et al. 2023). The concept of all‐on‐four treatment offers several clinical advantages: as the need for demanding bone grafts can be eliminated, there is less surgical morbidity and postsurgical discomfort (Rosén and Gynther 2007), improved load distribution due to shorter prosthetic cantilevers (Peñarrocha‐Oltra, Covani, et al. 2013), reduced number of fixtures for full‐arch prosthesis (Babbush et al. 2014), and decreased postsurgical discomfort (Aparicio et al. 2001). Most importantly, this approach offers the advantage of immediate loading, thus providing immediate rehabilitation of the maxilla with minimized costs and maximized patient satisfaction (Malo et al. 2012; Babbush et al. 2013; Gonçalves et al. 2022). However, current evidence regarding the survival rate of these implants inserted in pristine bone compared to those placed in grafted bone is limited by the short follow‐up periods.

This study assessed the survival rate of implants that support fixed full‐arch maxillary prostheses in patients with moderate to severe alveolar atrophy, treated with graftless (GL) and grafted (GG) approaches. The preliminary up to 10‐year outcome did not show a clinically significant difference between the two study groups, regardless of the implant loading protocols. Therefore, our null hypothesis stating that no significant difference exists in the implant survival rates of the graftless and grafted groups was accepted.

Clinical evidence directly comparing implant survival rates of the graftless and grafted approaches for atrophic edentulous maxillae is lacking, since previous studies comparing the two approaches only reported shorter follow‐up times (Malo et al. 2012; Testori et al. 2008; Calandriello and Tomatis 2005). To our knowledge, this is the first long‐term cohort study comparing the outcome of dental implants placed in grafted bone vs native bone in the atrophic edentulous maxillae using two different loading protocols.

In our study, the survival rate in the graftless group and for tilted implants was 98.4% (Table 2 and Figure 3B,D), as only two implants failed from a total of 124 tilted graftless implants, which is in accordance with previously reported results (Testori et al. 2008; Calandriello and Tomatis 2005; Babbush et al. 2011). In this line, Maló et al (Maló et al. 2005) reported a 5‐year survival rate estimation of 97% and 98.8% at patient and implant level, respectively, and most recently, Agliardi et al. (Agliardi et al. 2023) showed a cumulative implant survival rate of 97.51% for the maxilla, suggesting that two axial and two tilted implants could be considered a viable treatment in the long‐term.

For that matter, in our study, no statistically significant differences were found between tilted and axial implants within the graftless group (GL group; Table 3, Figure 3B), which is in agreement with the most recent studies (Agliardi et al. 2023; Hopp et al. 2017). In this line, Chrcanovic et al. (Chrcanovic et al. 2015) in a meta‐analysis that included 44 publications, resulting in a total of 5029 tilted and 5732 axial implants reported similar survival rates for tilted and axial implants, demonstrating as well that tilted implants in the immediate rehabilitation are safe and not associated with a higher marginal bone loss. In fact, combining straight and tilted implants seems to improve bone anchorage and primary stability as the surface of osseointegration increases (Ata‐Ali et al. 2012) and may be effective for stress distribution since cantilever extremities can be avoided (Karimov et al. 2024). However, in a recent meta‐analysis, tilted implants have shown a greater risk of marginal bone loss than axial implants when supporting fixed partial dentures (Batista et al. 2024).

**Table 3:** Implant survival analysis according to implant‐based covariates.

	Successful implants	Failed implants	Hazard ratio (95% CI)	*p*‐value
*N implants/n patients*	390/78	9/9		
Implant site (*n* implants)				0.147^a^
GL group	240	4	0.18 (0.02–1.81)	
GG group	150	5	1	
Implant diameter			1.13 (0.05–26.95)	0.938^a^
Mean (SD)	4.03 (0.12)	4.07 (0.13)		
[95% CI]	[4.02–4.04]	[3.98–4.15]		
Implant Length (mm)			0.82 (0.57–1.18)	0.280^a^
Mean (SD)	12.44 (1.86)	11.78 (1.95)		
[95% CI]	[12.26–12.63]	[10.50–13.06]		
Implant Torque (N·cm)			1.02 (0.96–1.07)	0.578^a^
Mean (SD)	32.04 (13.26)	32.51 (16.48)		
[95% CI]	[30.71–33.36]	[21.71–43.31]		
Implant Angulation (*n* implants)				0.227^a^
Tilted	124	2	3.61 (0.45–28.95)	
Straight	266	7	1	
Implant location (*n* implants)				0.401^a^
Anterior	175	5	0.45 (0.07–2.94)	
Posterior	215	4	1	
Implant number			0.805 (0.40–1.64)	0.748^a^
Mean (SD)	4.79 (1.08)	5.11 (1.05)		
[95% CI]	[4.70–4.90]	[4.42–5.80]		
Opposing arch (*n* implants)				< 0.001^a^
RCD/overdenture	23	0	1	
Natural dentition/IS‐FPD	305	5	3.58e^8^ (5.95e^7^−2.13e^9^)	
IS‐FFA	62	4	1.54e^9^ (−)	

Abbreviations: IS‐FFA, implant‐supported fixed full‐arch prostheses; IS‐FPD, implant‐supported fixed‐partial denture; RCD, removable complete denture.

*Estimate of the ratio of the hazard rate in the GL versus GG group.

^a^ *p*‐value calculated using the Cox proportional hazards model with clustered data.

**Table 4:** Implant survival according to patient‐related covariates.

	Successful implants	Failed implants	Hazard ratio (95% CI)	*p*‐value
*N* implants/*n* patients	390/87	9/9		
Age (years), mean (SD), [95% CI]	60.07 (8.50)	59.73 (14.19)	1.03 (0.90–1.17)*	0. 695^a^
	[59.22–60.92]	[48.82–70.63]		
Follow‐up period (months), mean (SD), [95% CI]	96.00 (29.72)	38.52 (37.34)		< 0.001^b^
	[93.05–98.96]	[9.81–67.22]		
Sex (implant‐based)				0.595^a^
Male	175	4	1.54 (0.31–7.62)	
Female	215	5	1	
Smoking habits	49	1		0.338^a^
Nonsmoker	347	8	1	
Smoker	43	1	3.10 (0.31–31.55)	

* Estimate of the ratio of the hazard rate in the GL versus GG group.

^a^ *p*‐value calculated using the Cox proportional hazards model with clustered data.

^b^ *p*‐value calculated using Student *t‐*test, equal variances not assumed.

Meanwhile, in the grafted group, the overall implant survival rate was 96.8% (Figure 3A; Table 2). In this line, a recent systematic review of 40 studies showed an overall weighted mean implant survival rate in augmented edentulous maxillary bone of 85.2% for onlay bone grafting, 91.5% for the sinus augmentation technique, and 93.6% for the combination technique (Aghaloo et al. 2017); however, studies on implants placed using the graftless approach for management of the atrophic maxilla have shown relatively higher implant survival rates (Jemt and Johansson 2006; Chrcanovic et al. 2015).

Failure generally occurred within the first year and was related to clinical complications, such as recurrent acute and chronic sinusitis. Regarding the nature of the implant failures observed in our study, two of the five implants lost in the grafted group were in patients with no systemic diseases that presented an insertion torque of 17.6 and 20 N·cm, respectively. This relatively low torque could have played a role in their failure since avoiding micromovements and achieving primary stability are crucial for the osseointegration process. However, a consensus is lacking on adequate insertion torque values during implant placement, as other clinical studies have shown that a low insertion torque (less than 30 N·cm) is enough for implant survival, regardless of the treatment protocol (Lemos et al. 2021; Trisi et al. 2009). The three other failures in the grafted group occurred in patients with a history of depression, previous oncogenic process, hypertension, and treated with antidepressants, antihypertensive drugs, and PPIs. Moreover, two out of five failed implants in the grafted group occurred in patients with diabetes and hypothyroidism. These systemic conditions and medications could have played a role in increasing the risk of implant failure. Indeed, many studies have suggested that chemotherapy, PPIs, and antidepressants can hinder bone healing and impair implant osseointegration (Abu Nada et al. 2018; Mahri et al. 2021; Chappuis et al. 2018). Furthermore, diabetes type‐2 might be responsible for the failure for the two above‐mentioned failures, as it has been suggested as a possible risk factor for implant failure due to poorly controlled glycemic levels (Bencze et al. 2024).

From the results of our study, it can be suggested that the nature of the opposing arch may play a role in the survival rate of implants, since the opposing arch status showed a statistically significant association with the implant failure (*p* < 0.001). In fact, implant failures only occurred when the opposing arch was natural dentition or implant‐supported fixed partial prostheses (60% of implant failure), or implant‐supported fixed full‐arch prostheses (40% of implant failure) (Table 3). Our findings are in accordance with a growing body of evidence that suggests that higher occlusal forces are generated by patients with a fixed complete denture or natural dentition (Ventura et al. 2016; Eliasson et al. 2000; Mackert et al. 2022; Müller et al. 2012). This higher implant failure rate may be explained by the increased occlusal forces when compared to edentulous jaws (Eliasson et al. 2000; Mackert et al. 2022; Müller et al. 2012), the lack of fine proprioception, and lower occlusal tactile sensitivity (Davis et al. 2003; Grieznis et al. 2010) and the parafunctional loading associated with fixed implant rehabilitations (Zierden et al. 2021; Karre et al. 2023).

Conversely, in our study, no implant failure was observed when the opposite arch consisted of a removable restoration, which is most likely because a removable denture can help transfer occlusal loads over a larger area, generating uniformly distributed masticatory forces (Müller et al. 2012). Although both grafted (96.8%) and graftless treatments (98.4%) presented very high implant survival rates, the conventional grafting approach required a much longer waiting time leading to the delivery of the fixed functional prosthesis (19.13 ± 6.91 months; Table 2) compared to graftless approach (0.63 ± 1.9 months; *p* < 0.001; Table 2). Furthermore, in our study, the graftless procedure (GL group) required fewer implants (mean 4.6 ± 0.9, Table 2) in comparison to the grafted approach, where a mean of six implants were placed (6.1 ± 0.7; *p* < 0.001; Table 2). These differences in treatment length, morbidity, and economic costs are most likely to have an important impact on patients’ quality of life (Calandriello and Tomatis 2005; Ata‐Ali et al. 2012), especially during the transitional period favoring the graftless approach (Ata‐Ali et al. 2012). However, this would need to be investigated in future studies designed to provide a more comprehensive analysis of the economic and life implications of this approach.

The uniqueness and strengths of this cohort study were that all the surgical and restorative procedures were completed by the same surgeon and the same prosthodontist, respectively, thus eliminating personal and operational variations. However, this study had several limitations. The group size difference may have masked the conclusion that there was no difference in implant survival between the grafted and graftless approaches. Furthermore, many significant cofounding variables that affect the implant success, such as peri‐implant health, bruxism, and history of periodontal disease, were impossible to assess given the study design and its retrospective nature. Moreover, we could not determine implant success; instead, we investigated implant survival, which could undermine the generalizability of our findings. Implant success allows for a much deeper understanding of implant performance; nevertheless, this additional information could not be retrieved from the patients’ records. Moreover, the absence of standardized peri‐apical radiographs or customized radiographic jigs did not allow for exact quantitative measurements of marginal bone loss and apico‐coronal positioning. Nonetheless, according to well‐established bone assessment methods (Hopp et al.2017), CBCTs were used to determine bone loss. Another limitation was the relatively small sample size, the fact that the study was performed in a single center, and the shortcomings owing to the retrospective nature of this study (Talari and Goyal 2020).

Despite the above‐mentioned limitations, the small number of failures combined with the long follow‐up period of our current study highlights the validity of the graftless treatment concept of the atrophic maxilla as an alternative to the conventional grafting approach.

## 5 Conclusions

Within the limitations of this up to 10‐year retrospective cohort study, it can be suggested that:

1. Immediately loading resorbed edentulous maxillae with axial and tilted standard implants using a graftless approach provides an implant survival rate that is not significantly different from that achieved with the conventional grafted approach.
2. The time‐to‐function in the graftless group is significantly shorter than that of the conventional grafted group.
3. The nature of the opposing arch may play a role in the survival rate of implants.

## Author Contributions

**Evelina Haroyan‐Darbinyan:** data analysis and interpretation, writing – original draft, review, and editing. **Qiman Gao:** statistics, writing original draft. **Pablo de Lillo:** statistics, data analysis/interpretation**. Jesus Torres:** validation, supervision, conceptualization. **Faleh Tamimi:** study design, supervision, visualization, validation, methodology, project administration, review, and editing. **Abi Nader Samer:** supervision, visualization, data collection, validation, conceptualization, project administration, writing original draft, and editing. **Daniel Nach:** data collection and methodology.

## Ethics Statement

This retrospective cohort study was approved by the Research Ethics Committee for Clinical Trial of McGill University (Reference number: 12‐321 GEN).

## Consent

Written informed consent was obtained from all patients.

## Conflicts of Interest

The authors declare no conflicts of interest.

## Data Availability

The data that support the findings of this study are available on request from the corresponding author. The data are not publicly available due to privacy or ethical restrictions.
